# Computed tomographic appearance of transcaval ureter in two dogs and three cats: A novel CVC congenital malformation

**DOI:** 10.3389/fvets.2022.965185

**Published:** 2022-09-08

**Authors:** Carlotta Spediacci, Maurizio Longo, Swan Specchi, Pascaline Pey, Silvia Rabba, Eirini Mavraki, Mauro Di Giancamillo, Ioannis Panopoulos

**Affiliations:** ^1^Department of Veterinary Medicine and Animal Science (DIVAS), University of Milan, Lodi, LO, Italy; ^2^Diagnostic Imaging Department C.T.O. Veterinary, via C. Festa Arenzano (Genova), Genova, Italy; ^3^Antech Imaging Services, Irvine, CA, United States; ^4^Ospedale Veterinario I Portoni Rossi, Bologna, Italy; ^5^Service d'Imagerie Médicale, Centre Hospitalier Universitaire Vétérinaire d'Alfort, Ecole Nationale Vétérinaire d'Alfort, Maisons-Alfort, France; ^6^Department of Diagnostic Imaging, Istituto Veterinario di Novara, Granozzo con Monticello, NO, Italy; ^7^Vets4life Veterinary Clinic, Pikermi, Athens, Greece; ^8^Alphavet Veterinary Diagnostic Imaging, Kifisia, Athens, Greece

**Keywords:** peri-ureteral venous ring, caudal vena cava malformation, canine, feline, imaging

## Abstract

Transcaval ureter is a rarely reported human congenital malformation of the prerenal segment of the inferior vena cava (IVC) not yet reported in veterinary medicine. The objective of this multicenter retrospective case series study was to describe the computed tomography (CT) features of transcaval ureters in dogs and cats. Patients referring to pre- and post-contrast CT exams of the abdomen and presenting this abnormality were retrospectively included. Multiple qualitative features were described for each ureteral abnormality detected. Three cats and two dogs with transcaval ureter were identified consisting of a segmental duplication of the CVC at the prerenal level creating a vascular ring through which the ureter extended, identified as a *double-barrel gun* sign. The malformation was divided into two types according to the symmetry of the caval branches and location in relation to the aorta, namely, type I symmetrical branches and right-lateral to the aorta, and type II asymmetrically branches and right-dorsal to the aorta. In one case, the malformation was associated with hydroureter and mild pyelectasis. In three cases, the anomaly was incidental and, in the remaining two cases, the clinical significance was uncertain. This is the first study describing the presence of transcaval ureter in dogs and cats. CT was a suitable method for the diagnosis of transcaval and a focal *double-barrel gun* sign of the CVC is proposed as the hallmark feature of this anomaly. The clinical relevance of this congenital vascular malformation is unclear and needs to be further investigated.

## Introduction

Variation in the anatomy of caudal vena cava (CVC) may have important clinical implications. In-depth knowledge of its anatomy is fundamental to prevent any potential medical and surgical complications related to the anatomical variation of the CVC (1–4). Normal embryogenesis of the CVC leads to the origin of a single vessel positioned to the right of the aorta artery (4, 5). It is created by persistence, regression, and anastomosis of three bilateral symmetrical embryonic veins, namely, the postcardinal, the subcardinal, and supracardinal. An anomaly in the development of these vessels results in congenital vascular anomalies of the CVC (4–7). In veterinary medicine, several well-recognized congenital anomalies of the CVC have been reported, including double CVC and retrocaval ureter. Contrast-enhanced multidetector computed tomography (MDCT) for detecting and characterizing CVC anomaly is recommended due to the excellent visualization of the CVC and its relationship with the ureters (5). Transcaval ureter is a rare embryological abnormality of the inferior vena cava (IVC) rarely described in human medicine, in which a segmental duplication of this vessel creates a venous ring that encircles the right ureter (8–11). There are few reports on transcaval ureter in human medicine, and different theories about its embryological development were formulated (2, 8–11). According to the supracardinal model, transcaval ureter arises when the right posterior cardinal and right supracardinal veins fail to regress in post-renal segment, resulting in a partial duplication of IVC that creates a venous ring through which the right ureter extends. The reported prevalence of transcaval ureter in humans is about 0.9 in 1,000 with a male-to-female ratio of 2.8:1 (12). This abnormality in human patients is described as often asymptomatic but can sometimes result in flank pain, hydroureteronephrosis, recurrent urinary infections, increased frequency of urination, and hematuria (13–15).

To the best of our knowledge, this anomaly and the CT features of the transcaval ureter have never been reported in veterinary medicine. The aim of the study was to describe the CT characteristics of transcaval ureters in case series dogs and cats.

## Materials and methods

This was a retrospective, descriptive, multicenter, case-series design study. Clinical cases from different institutions (e.g., I Portoni Rossi, University Veterinary Hospital d'Alfort, Istituto Veterinario di Novara, C.T.O. Veterinario, and Alphavet Clinic) were searched for dogs and cats with a diagnosis including the keywords “atypical CVC malformation.” Permission to use clinical and diagnostic imaging data for research was provided through informed consent from the owners at the time of admission. The inclusion criteria of this study were a final diagnosis of unclassified vascular malformation consisting of a venous ring encircling the ureter and originating from the CVC and an available CT study of diagnostic quality of the abdomen before and after intravenous contrast medium injection. Patients were included regardless of sex, breed, age, clinical signs, and acquisition parameters or scanner types (16 and 64 slices). The decisions for study inclusion were based on a common consensus between all authors. Medical records were reviewed regarding species, age, breed, sex, body weight, clinical signs, and final diagnosis. Pre- and post-contrast images were stored on a dedicated picture archiving and communication system (PACS) and evaluated by four (PP, IP, ML, and SR) ECVDI and one (SS) ACVR board-certified veterinary specialists. All assessments were performed using the dedicated DICOM viewer software (OsiriX Imaging Software, version 3.9.2., Pixmeo, Geneva, Switzerland). A soft tissue window width of 350 Hounsfield unit (HU) and a window level of 50 HU were used. In all cases, the images were reconstructed in multiple reformatted planes and on 3-D, if needed. The segmental bifurcation of the CVC in cross-section assumed the appearance of two adjacent round structures that have been defined as “*double-barrel gun sign*.” The following parameters of the CT scan were recorded: position of the venous ring in relation to aorta and lumbar spine, diameter of the ureter both proximal and distal to the venous ring expressed in millimeters, presence of pyelectasis, thrombosis, and concomitant congenital malformation. Ureteral dilation, if present, was diagnosed when the maximal diameter of the cross-section of the ureter was measured more than 2.5 mm in dogs and cats (16, 17). Pyelectasis was diagnosed when the maximal diameter of the renal pelvis was measured more than 3 mm in both dogs and cats. Renal pelvis > 6 mm was defined as suggestive of suspected mechanical obstruction, while renal pelvis > 13 mm was defined as unequivocally obstructed (18–20). All assessments were based on visual inspection. CT images were assessed blindly, without the knowledge of the signal and clinical findings from the patients. If there were different opinions about CT findings, a final diagnosis was made based on the consensus of all the observers. Additional unrelated CT findings were recorded, when observed.

## Results

### Case 1

**Figure 1:**
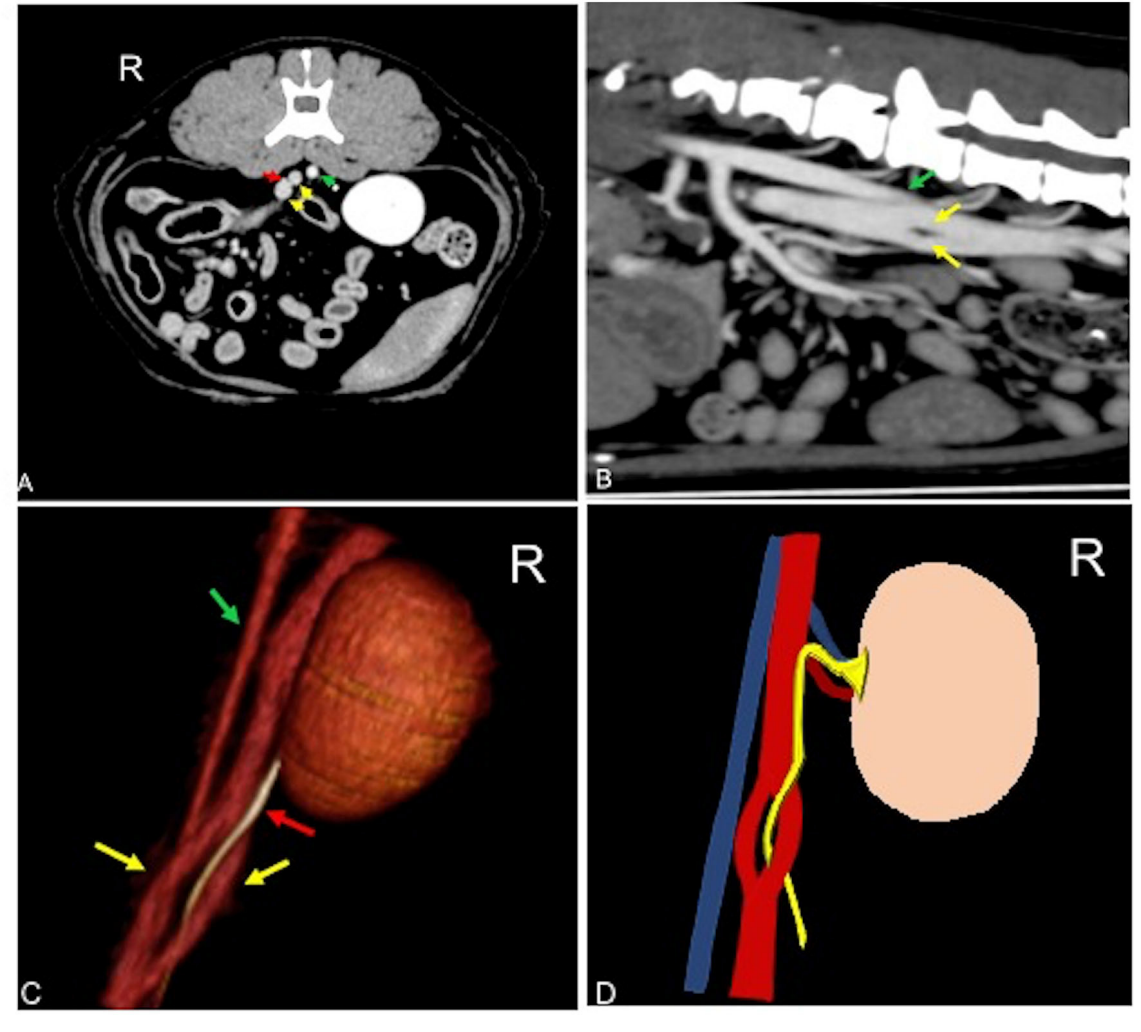
Postcontrast computed tomographic of mid abdomen in transverse **(A)**, sagittal **(B)**, volume rendering image in a right-dorsal view **(C)**, and schematic representation of the malformation **(C)** showing the CVC venous ring (yellow arrow) described as double barrel-gun sign with the right ureter (red arrow) passing through it. The venous malformation is normally located right-ventral to the aorta (green arrow). Soft tissue reconstruction (window level: 40 HU, window width 400 HU) and a slice thickness of 1.25 mm were used. In **(D)**, the aorta is blue, the CVC is red, and the ureter is yellow. R, right.

An 11-year-old intact female domestic shorthaired cat was presented to Instituto Veterinario Novara for a whole-body pre- and post-contrast CT exam for the staging of mammary nodules. No clinical signs referable to anomalies of the urinary tract were reported. Blood biochemistry and urinalysis were normal. Urine culture was not available. CT revealed a unilateral transcaval ureter, in which the right ureter emerged through a venous ring formed by a segmental duplication of the CVC at the level of L4–L5. The two branches of the CVC were symmetrical in diameter with a *double-barrel gun* appearance and were normally located right-ventral to the aorta (Figure 1). The diameter of the ureter both proximal and distal to the venous ring measured normal (0.6 mm) (17, 21). Other CT findings were mammary nodules associated with inguinal lymphadenopathy and bilateral renal infarcts. There was no evidence of thrombosis, pyelectasis, ureteral dilation, or other concomitant congenital malformation.

### Case 2

**Figure 2:**
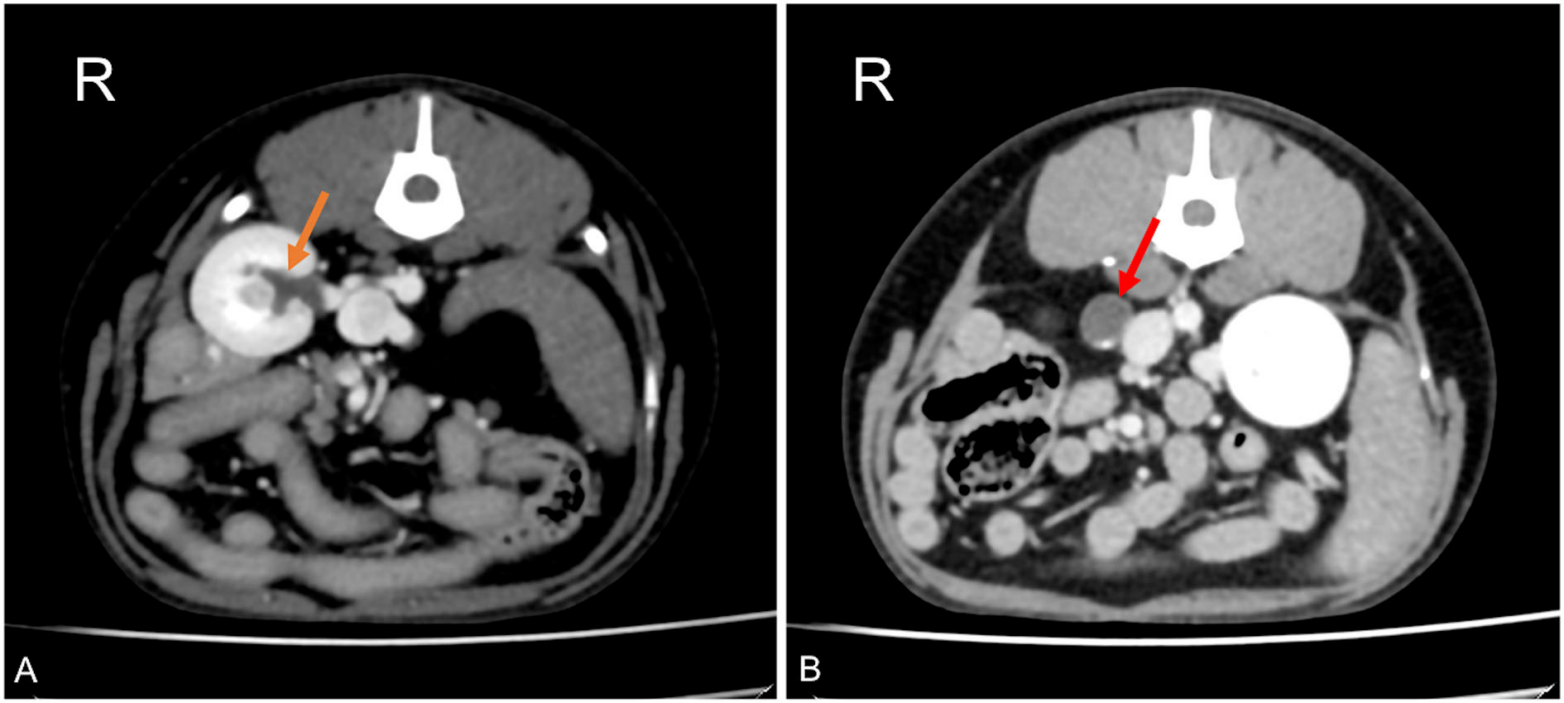
Postcontrast computed tomographic images of mid abdomen in transverse views at the level of the right kidney **(A)** and just proximally to the venous ring **(B)** in a dog showing dilation of the renal pelvis (orange arrow) and dilation of the right ureter proximally to the venous ring malformation (red arrow). Soft tissue reconstruction (window level: 40 HU, window width 400 HU) and a slice thickness of 1.25 mm were used. R, right.

A 4-year-old Maltese neutered male dog was presented to Alphavet Diagnostic Imaging Center for an abdominal pre- and post-contrast CT exam and a 4-month history of recurrent vomiting. The patient did not show improvement after medical treatment consisting of metoclopramide and ranitidine performed at the referring veterinarian. Blood biochemistry showed renal enzymes within normal ranges, while biliary acids were slightly increased at 4.9 μmol/L (normal range of 0.2–4.3 μmol/L). Urine culture was not available. The referring veterinarian suspected chronic hepatitis. CT revealed a unilateral transcaval ureter, in which the right ureter emerged through a venous ring at the level of L3–L4. The two branches of the CVC were symmetrical in diameter with a *double-barrel gun* appearance and were normally located right-ventral to the aorta. The ureter proximal to the venous ring was homogeneously distended (diameter of 9.6 mm) with associated mild pyelectasis (3.4 mm) (Figure 2) (18–20). Distal to the venous ring, the ureter measured normal (1.4 mm) (16). There was no evidence of thrombosis or other concomitant congenital malformation.

### Case 3

A 6-year-old medium-sized mixbreed neutered female dog was presented to the C.T.O. Veterinario for abdominal pre- and post-contrast CT exam to investigate the causes of hematuria. Blood biochemistry and urine culture were normal. On urinalysis, the urine specific gravity (USG) was 1.100, pH 6, and blood 1+. Urine sediment revealed the mild presence of red blood cells. On CT examination, a right transcaval ureter was found. CT revealed a unilateral transcaval ureter, in which the right ureter emerged through a venous ring at the level of L3–L4. The two branches of the CVC were symmetrical in diameter with a horizontal *double-barrel gun* appearance and were normally located right-ventral to the aorta. Proximal and distal to the venous ring, the right ureter measured normal (1.7 mm) (16). There was no evidence of thrombosis, pyelectasis, ureteral dilation, or other concomitant congenital malformation.

### Case 4

A 2-year-old domestic shorthaired neutered female cat was presented to the Centre Hospitalier Universitaire Vétérinaire d'Alfort for a whole-body pre- and post-contrast CT exam. The patient had a history of a car accident 24-h before the presentation. The patient presented with pelvic fractures and an absence of deep pain in the hind limbs. No clinical signs referable to anomalies of the urinary tract were reported. Blood biochemistry and urinalysis were normal. Urine culture was not performed. CT revealed a unilateral transcaval ureter, in which the right ureter emerged through a venous ring at the level of L4–L5. The two branches of the CVC were symmetrical in diameter with a *double-barrel gun* appearance and were located lateral to the aorta (Figure 1). The diameter of the right ureter both proximal and distal to the venous ring was slightly increased (0.4 mm) (16). Other CT findings consisted of comminuted and displaced nonarticular closed traumatic fractures of the pubis and right ileus with concomitant left sacroiliac luxation. There was no evidence of thrombosis, pyelectasis, or other concomitant congenital malformation.

### Case 5

**Figure 3:**
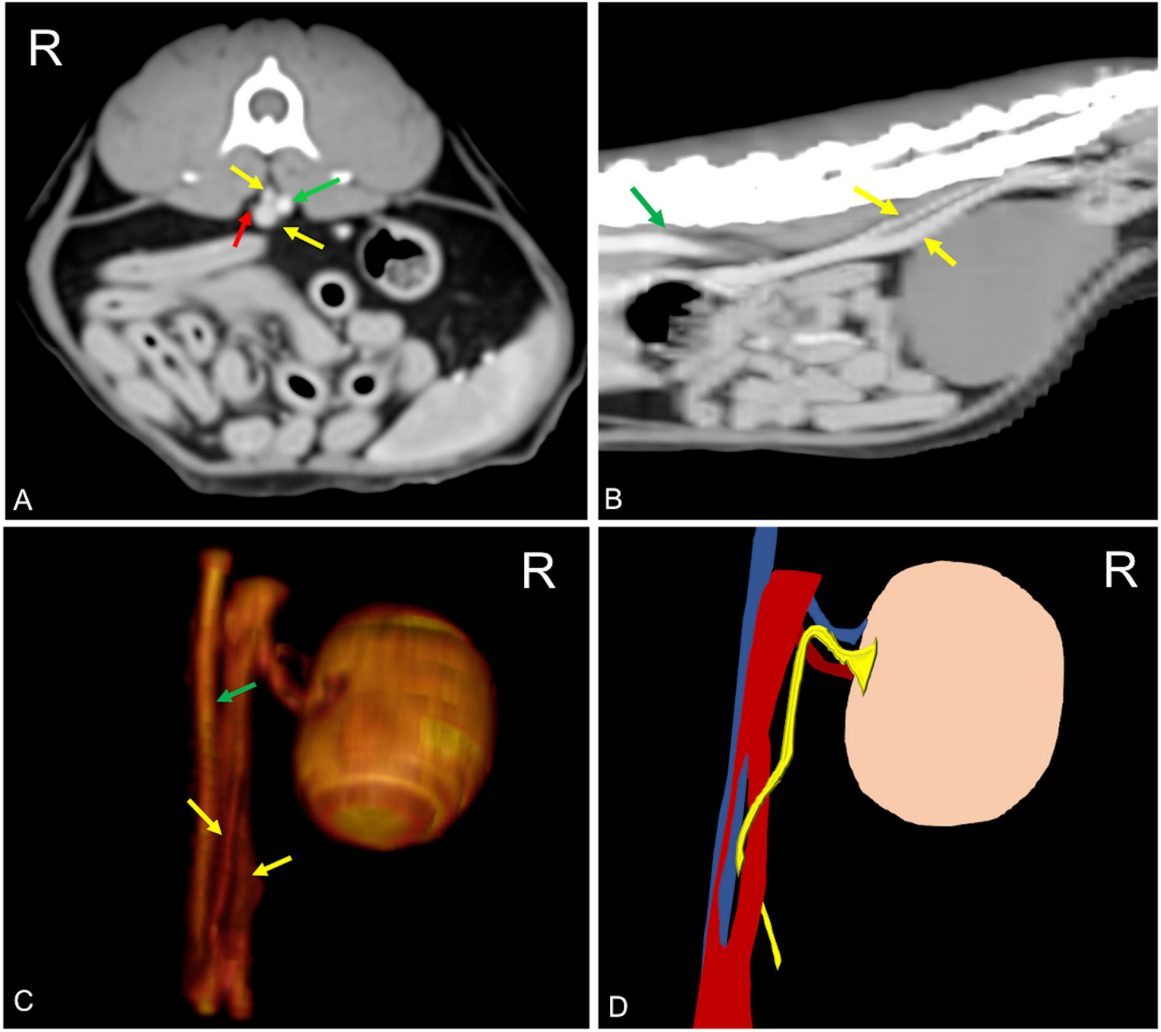
Postcontrast computed tomographic of mid abdomen in transverse **(A)**, sagittal **(B)**, volume rendering image in a right-dorsal view, **(C)** and schematic representation of the malformation **(C)** showing the CVC venous ring (yellow arrow) described as double barrel-gun sign with the right ureter (red arrow) passing through it. The two branches of the venous ring appear asymmetric with the dorsal branch located dorsally to the aorta (green arrow). Soft tissue reconstruction (window level: 50 HU, window width 350 HU) and a slice thickness of 2.5 mm were used. In **(D)**, the aorta is blue, the CVC is red, and the ureter is yellow. R, right.

A pre- and post-contrast CT exam of the thorax, abdomen, and spine of a 5-year-old domestic shorthaired neutered female cat was submitted as a teleradiology consult to Antech Imaging Services. The referred clinical signs of the patient are consistent in acute onset of pelvic limb ataxia. Blood biochemistry and urinalysis were normal. Urine culture was not performed. CT findings consisted of the presence of a pulmonary mass, while the spine appeared unremarkable. In the abdomen, a unilateral transcaval ureter, in which the right ureter emerged through a venous ring extending from L3 to L6, was detected. The two branches of the duplicated CVC were asymmetrical with a *double-barrel gun* appearance, in which the dorsal branch had a smaller diameter, and it was positioned right dorsal to the aorta (Figure 3). Diameter of the right ureter both proximal and distal to the venous ring was normal (0.3 mm) (16). There was no evidence of thrombosis, left hydronephrosis, ureteral dilation, or other concomitant congenital malformation. In this patient, duplication of the spleen was also detected. Other findings consisted of left psoas muscle changes and jejunal lymphadenopathy. There was no evidence of thrombosis and pyelectasis.

## Discussion

To the best of our knowledge, this is the first case series describing the presence and the CT appearance of transcaval ureter in dogs and cats. In all cases described in this study, the transcaval ureter was unilateral to the right and appeared as a vascular ring of the CVC through which the right ureter passed identified as *double barrel-gun sign*. In only one case, hydronephrosis and mild pyelectasis were associated (Table 1).

**Table 1:** Demographic details of patients included in the study with qualitative characteristics of the venous ring malformation.

**N**	**Species**	**Breed**	**Sex**	**Age**	**Location of venous ring**	**Side of venous ring**	**Branches**	**Presence of hydroureter**	**Ureter diameter**
**1**	Cat	DSH	F	11	L4-L5	Right	Symmetrical	No	0.6 mm
**2**	Dog	Maltese	NM	4	L3-L4	Right	Symmetrical	Yes	9.6 mm
**3**	Dog	Mix breed	NF	6	L3-L4	Right	Symmetrical	No	1.7 mm
**4**	Cat	DSH	NF	2	L4-L5	Right	Symmetrical	No	0.4 mm
**5**	Cat	DSH	NF	5	L3-L6	Right	Asymmetrical	No	0.3 mm

DSH, domestic shorthaired cat; NM, neutered male; NF, neutered female; F, female; L, lumbar.

The CVC is composed of four major segments, namely, prerenal, renal, prehepatic, and posthepatic. The prerenal segment forms from the confluence of the common iliac veins to the right of the aortic trifurcation, along with the confluence of the right gonadal vein. The renal segment forms ventrally and to the right of the abdominal aorta, from the union of the right and left renal veins with the left gonadal vein. Finally, the prehepatic, hepatic, and posthepatic course forms to the right dorsal portion of the liver (7, 22, 23). Various hypotheses have been formulated to explain the origins of the prerenal segment of the CVC. The most accepted theory is the “supracardinal model.” This theory assumes that the retroperitoneal venous system is developed from three paired fetal veins, namely, the posterior cardinal, the supracardinal, and the subcardinal veins. During embryological development of the CVC, the left supracardinal vein and the lumbar portion of the right posterior cardinal vein normally regress. The subcardinal vein becomes the internal spermatic vein and the right supracardinal vein develops into the definitive right CVC (23–25).

In accordance with the human literature, all dogs and cats included in this study reported a right-sided distribution of the transcaval ureter (8–11). Only patient number 3 had limited clinical signs related to the urinary system, consistent with hematuria which is also reported in human medicine (8, 9). All the patients included in this case series displayed normal kidney values. This is also similar to what is reported in people, as kidney function is unchanged despite possible gross changes in the ureter (2). For example, in cases of ureteral ectopia, the normal renal function is possibly related to the congenital origin of the anomaly and the adaptive process of the urinary tract during the development (26). Patient number 2 had intermittent vomiting despite absent gastrointestinal changes on CT. Flank pain might be considered in the differentials as a possible reason for the clinical signs, triggered by colic irradiation due to ureteral stretch, as reported in humans (9). However, a significant limitation regarding this hypothesis is that no additional procedures, such as gastrointestinal endoscopy and biopsy, were performed to further investigate the cause of vomiting. A moderate right hydroureter and mild ipsilateral pyelectasis were also detected in patient number two. This has some similar characteristics to retrocaval ureter type I (“fishhook deformity”). Nevertheless, in this type of retrocaval ureter, there is an extreme medial deviation of the middle ureteral segment where it passes behind the IVC/CVC, usually at the L3 vertebral level (3). In our case, the deviated portion of the ureter splits the CVC and, therefore, courses gently medially rather than in a typical type I retrocaval ureter. In addition, the level of mechanical obstruction due to the vascular ring was located more caudally compared to a typical retrocaval ureter. Although the pelvic dilation was mild, the guidelines of the American College of Veterinary Internal Medicine (ACVIM) indicate that hydronephrosis and hydroureter proximal to an obstructive lesion are sufficient to diagnose mechanical obstruction (17). Therefore, in this patient, ureteral obstruction is likely caused by the vascular malformation. However, due to the absence of clinical signs and normal renal parameters, further treatment and investigations were discarded. Hydroureter and pyelectasis may predispose to recurrent urinary tract infection. In human medicine surgical management is recommended in case of complications, including recurrent urinary infections, urolithiasis, or impaired renal function (27). In patient number one, renal infarcts were found on the CT examination. Kidney infarction is caused by sudden occlusion of blood flow to a renal pyramid. Renal infarcts can develop due to multiple diseases that lead to an increase in thrombus formation, such as hyperthyroidism, neoplasia, or cardiomyopathy (28). In humans, anomalies of the IVC have been reported to be a risk factor for the development of deep vein thrombosis. However, this has not been described in veterinary medicine; therefore, a correlation between renal infarcts and transcaval ureter should be considered cautiously (27, 29–33).

In human and veterinary medicine, congenital anomalies of the IVC/CVC are increasingly recognized in asymptomatic patients due to the increased use of cross-sectional imaging technique such as CT, offering simultaneous evaluation of the vascular system and urinary tract with the aid of uroangiographic ICM (30). Although the anomalies of the vessels and ureters are often clinically silent, it is important to recognize them in order to avoid potential complication during surgery and interventional radiology procedures (31). In humans, CT angiography provides precise preoperative information about abdominal vascular anatomy, with a reported accuracy of >97% for arteries and 96–100% for veins (31).

Transcaval ureter may show similarities with retrocaval ureter, in which the ureter run medially to the CVC and, therefore, should be considered in the differential diagnosis and diagnostic process (10). Retrocaval ureter occurs as a result of abnormal persistence of the right cardinal vein, which is crossed dorsally and medially by the ipsilateral ureter. A prevalence of 22.4% was reported with the right kidney involved 13.3% times more frequently than the left (5, 29). Two types of retrocaval ureter are described in human medicine and the same classification has been used in veterinary medicine, namely, type I, or low loop, and type II, or high loop (34–36). Type 1 is described as a possible cause of ureteral obstruction, while type 2 is associated with mild or no hydronephrosis and occurs in only 10% of patients (34). Transcaval ureter is not susceptible to this specific classification despite showing characteristics between these two types (1). A focal *double-barrel gun* sign of the CVC is proposed as the hallmark sign of the anomaly, which should be recognized and differentiated from a common retrocaval ureter. A clear and direct identification of the defect can only be achieved by a late venous post-contrast CT exam, allowing simultaneous visualization of the CVC and ureter. Nevertheless, detection of the defect can be challenging and may require multiplanar or 3-D multiplanar reconstructions.

In human medicine, surgical management involves resection of the ureter and an ureteroureteric anastomosis anterior to the IVC (9). To the best of our knowledge, there is no surgical procedure described in veterinary medicine for the treatment of a transcaval ureter since this congenital malformation has never been described and its clinical impact is still uncertain. However, division of the dilated renal pelvis with transposition and anastomosis, ureterotomy or resection of the stenotic segment of the ureter compressed by the anomalous vessel with anastomosis of a double-J stent, and ligation or transection of the CVC with or without anastomosis are also procedures indicated in case of renal obstruction (34, 37).

The association of congenital anomalies of the IVC/CVC with other malformations is reported in both human and veterinary medicine, but the incidence is still unclear because these anomalies might be often underdiagnosed. Small-breed dogs was reported that 7% of patients with malformation of CVC might present with a concomitant extrahepatic portosystemic shunt, while a left-sided circumcaval ureter was reported in association with the transposition of the CVC (5, 30, 32). In the present case series, the only concomitant congenital anomaly found was duplication of the spleen in one patient; however, for the authors knowledge, this anomaly has never previously been associated with IVC/CVC malformations (37).

In this case series, two vascular patterns of the transcaval ureter have been described. The first type consisted of a *double-barrel gun* appearance with symmetrical vascular branches, normally located right-ventral to the aorta. The second type consisted of a *double-barrel gun* sign with asymmetrical vascular branches, located right dorsal to the aorta. Previous studies have reported that asymmetry of venous diameters in cases of CVC duplication is secondary to a regression disorder of the supracardinal vein, which may be located dorsolaterally to the aorta in the caudal portion of the prerenal segment (30, 32). Despite this theory was formulated for the duplication of IVC/CVC, the authors speculate that similar embryogenetic processes may also be involved in generating this particular pattern of congenital malformation.

Multidetector angiographic CT exam in all cases demonstrated to be a valid technique in the diagnosis of transcaval ureter allowing simultaneous assessment of ureters and CVC. In addition, techniques such as multiplanar and three-dimensional reconstruction, including volume rendering, can be used to further investigate the morphology of the defect. In particular, multiplanar reconstruction can assist in understanding the anatomy and the relationship between the ureter and the vascular ring.

The limitations of this study are related to the retrospective and multicentric design. Indeed, CT exam parameters and scanners were not standardized and the quality of the urographic excretory phase was not standardized either. Furthermore, there was inconsistency of data collection and complete urinalysis, and additional examinations such as cystoscopy, pyelocentesis, or biopsy were not available in these series of patients. A further limitation is due to the retrospective data collection which might have affected the inclusion of additional cases.

In conclusion, transcaval ureter is a novel congenital malformation of the CVC in dogs and cats. The tomographic sign of transcaval ureter in both dogs and cats consists of a segmental duplication of the CVC at the prerenal level, creating a vascular ring through which the ureter extends, identified as a *double-barrel gun* sign. Further studies are needed in order to determine the clinical significance of this novel congenital malformation.

## Data availability statement

The raw data supporting the conclusions of this article will be made available by the authors, without undue reservation.

## Ethics statement

Ethical review and approval was not required for the animal study because it was not required because it was a retrospective descriptive case series study. Written informed consent was obtained from the owners for the participation of their animals in this study.

## Author contributions

CS, ML, and IP: designed the study and analyzed data. ML, SS, PP, SR, and IP: collected and complied data. CS: drafted this article. CS, ML, SS, PP, SR, EM, MD, and IP: revised the manuscript. All authors contributed to the article and approved the submitted version.

## Conflict of interest

Authors ML and IP were employed by company Alphavet Veterinary Diagnostic Imaging. Authors SS and PP were employed by Antech Imaging Services. The remaining authors declare that the research was conducted in the absence of any commercial or financial relationships that could be construed as a potential conflict of interest.

## Publisher's note

All claims expressed in this article are solely those of the authors and do not necessarily represent those of their affiliated organizations, or those of the publisher, the editors and the reviewers. Any product that may be evaluated in this article, or claim that may be made by its manufacturer, is not guaranteed or endorsed by the publisher.

## Abbreviations

CVC: caudal vena cava
IVC: inferior vena cava
CT: computed tomography
HU: Hounsfield unit
PACS: Picture Archiving and Communication System
L: Lumbar
IV: intravenous
ICM: intravenous contrast medium
MDTC: Multidetector Computed Tomography.
